# Acquired Hemophilia A: A Frequently Overlooked Autoimmune Hemorrhagic Disorder

**DOI:** 10.1155/2014/320674

**Published:** 2014-03-24

**Authors:** Yoshihiko Sakurai, Tomohiro Takeda

**Affiliations:** ^1^Department of Pediatrics, Matsubara Tokushukai Hospital, 7-13-26 Amamihigashi, Matsubara, Osaka 580-0032, Japan; ^2^Department of Clinical Laboratory Science, Kansai University of Health Sciences, 2-11-1 Wakaba, Kumatori, Osaka 590-0433, Japan

## Abstract

Acquired hemophilia A (AHA) is a rare hemorrhagic disease in which autoantibodies against coagulation factor VIII- (FVIII-) neutralizing antibodies (inhibitors) impair the intrinsic coagulation system. As the inhibitors developed in AHA are autoantibodies, the disease may have an autoimmune cause and is often associated with autoimmune disease. Although acute hemorrhage associated with AHA may be fatal and is costly to treat, AHA is often unrecognized or misdiagnosed. AHA should thus be considered in the differential diagnosis particularly in postpartum women and the elderly with bleeding tendency or prolonged activated partial thromboplastin time. Cross-mixing tests and measurement of FVIII-binding antibodies are useful to confirm AHA diagnosis. For treatment of acute hemorrhage, hemostatic therapy with bypassing agents should be provided. Unlike in congenital hemophilia A with inhibitors, in which immune tolerance induction therapy using repetitive infusions of high-dose FVIII concentrates is effective for inhibitor eradication, immune tolerance induction therapy has shown poor efficacy in treating AHA. Immunosuppressive treatment should thus be initiated to eradicate inhibitors as soon as the diagnosis of AHA is confirmed.

## 1. Introduction

During the course of treatment for autoimmune disease, patients with no history of bleeding sometimes suddenly present with severe ecchymoses or muscle hematoma. In such cases, acquired coagulation factor deficiencies, including acquired hemophilia A (AHA), should be considered in the differential diagnosis of the cause of bleeding [1]. As a rare hemorrhagic disorder but the most frequently acquired coagulation factor deficiency, AHA is caused by the development of antibodies, referred to as “inhibitors,” against coagulation factor VIII (FVIII), which neutralize FVIII activity. Although AHA has previously been reported to have an incidence of 0.2 to 1.0 cases per million population per year [2], a recent report describes a progressively increasing incidence of 2 cases per million population per year [3], likely resulting from greater awareness of the disorder. In contrast to the incidence of congenital hemophilia A, a recessive X-linked genetic disorder, the incidence of AHA has not been found to differ significantly between men and women. AHA has a biphasic age distribution, exhibiting a small peak from age 20 to 30 years and a larger peak at age 60 years and older [4, 5]. The majority of patients who present with AHA between ages 20 and 30 years are female, as the disease in this age group is associated with pregnancy (i.e., the development of postpartum inhibitors) and autoimmune disorders. While it was previously thought that the majority of patients who present with AHA at age 60 years and older are male [4, 6], recent studies have revealed no significant difference in the sex ratio of elderly patients [7].

While AHA has a high mortality rate, estimated at up to 33%, it has decreased in tandem with the advancement of therapeutic interventions since the 1980s [8]. AHA occurs relatively less frequently but develops suddenly and occasionally presents with life-threatening bleeding. Furthermore, the management of AHA remains difficult and the costs of treatment are often immense. Although AHA is thus clinically and economically an important disorder, it is often unrecognized or misdiagnosed as other acquired hemorrhagic disorders, such as disseminated intravascular coagulation (DIC) and acquired inhibitors against von Willebrand factor (acquired von Willebrand syndrome [9]) and factor XIII (acquired factor XIII deficiency [10]).

In contrast to the FVIII-neutralizing inhibitors that develop in congenital hemophilia A after FVIII-replacement therapy, which are alloantibodies, the FVIII-neutralizing inhibitors that develop in AHA are autoantibodies. It is well known that approximately 50% of patients with AHA have or have had immune system disorders, such as autoimmune diseases and lymphoproliferative disorders. This fact, as well as knowledge that autoantibodies play a central role in AHA pathogenesis, indicates that modulation of the immune system or the autoimmune mechanism that generates autoantibodies is involved in AHA.

## 2. Clinical Manifestations

AHA patients often present with severe and massive bleeding, which is responsible for their relatively high mortality rate. The most commonly affected organ is the skin, especially at the site of injection or contusion, which often manifests severe ecchymoses. Subsequently, intramuscular and gastrointestinal/intra-abdominal bleedings are often involved. It is notable that hemarthroses most commonly appear in congenital hemophilia A but seldom occur or cause joint damage in AHA [11, 12]. AHA is also associated with postdelivery or postoperative bleeding. Although relatively uncommon, intra-abdominal or intracerebral hemorrhage in AHA patients often leads to life-threatening bleeding. Persistent bleeding after surgical procedures, such as intramuscular injection, catheter insertion, and tracheotomy for treatment of underlying or incidentally coexisting diseases, may be the earliest symptom of AHA. Occasionally, AHA is suspected despite the absence of hemorrhagic manifestations by review of the preoperative examination results, especially in patients with low-titer inhibitors. A notable prognostic consideration is that, unlike in congenital hemophilia A, inhibitor titer in AHA does not indicate the severity or frequency of bleeding.

## 3. Characteristics of AHA Inhibitors

### 3.1. FVIII

**Figure 1:**
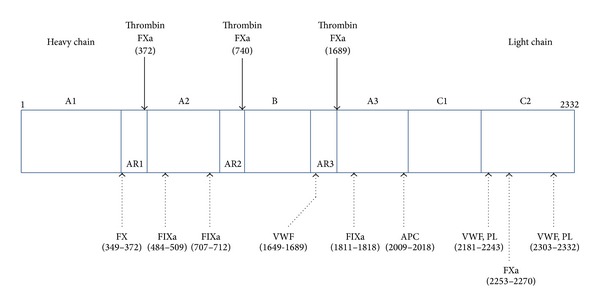
Structure of the coagulation factor VIII (FVIII) molecule. The numbers indicate amino acid positions. Plasma FVIII is a heterodimer composed of a heavy chain (domains A1, A2, and B) and a light chain (domains A3, C1, and C2). Noncovalent binding of FVIII with von Willebrand factor (VWF) protects circulating FVIII from being inactivated by activated protein C. The binding sites of VWF, phospholipids (PL), and other coagulation factors (activated factor IX [FIXa], factor X [FX], and activated FX [FXa]) are also indicated. FVIII is cleaved and activated by thrombin and FXa at residues 372 and 740 within the heavy chain and at residue 1689 within the light chain. Inhibitors impair FVIII activation by interfering with thrombin-catalyzed cleavage or FVIII interactions with VWF, FIXa, FX, and PL. AR: acidic region.

FVIII is a cofactor for activated factor IX (FIXa) that forms the Xase (tenase) complex in the presence of Ca^2+^ and phospholipids and is essential for the intrinsic coagulation system responsible for blood clotting; therefore, FVIII deficiency causes dysfunction of the intrinsic system and reduces thrombin generation, resulting in a bleeding disorder. FVIII is mainly synthesized in the liver as a 2,351 amino acid and 330-kDa single-chain precursor glycoprotein with a functional domain structure (A1-A2-B-A3-C1-C2) (Figure 1) [13]. After proteolytic processing, circulating mature FVIII protein is composed of a heterodimer of a heavy (A1-A2) and a light (A3-C1-C2) chain. This chain is noncovalently bound to von Willebrand factor (VWF), which protects the FVIII from inactivation. VWF has a molecular weight of 226 kDa and a multimeric structure consisting of subunits of large molecular weight (>20,000 kDa).

### 3.2. Characteristics of Inhibitors

The majority of FVIII inhibitors observed in AHA, which are polyclonal autoantibodies, and in congenital hemophilia A, which are polyclonal alloantibodies, bind to the A2 (454–509), A3 (1804–1819), or C2 domains (2181–2243) [14–17]. While anti-C2 antibodies interfere with the binding of FVIII to phospholipids and VWF, A2 and A3 inhibitors block the binding of FVIII to factor X (FX) and FIXa, respectively, and obstruct the formation of the Xase complex.

Previous studies of CD4 T-cell subsets (Th1, Th2, and Th3) specific for FVIII revealed that alloantibodies in congenital hemophilia A consist of Th1-dependent immunoglobulin (Ig) G1 and IgG2 and Th2-dependent IgG4. However, AHA autoantibodies are often IgG4 autoantibodies and less frequently IgG1 and IgG2 autoantibodies. Further, FVIII-neutralizing activity is correlated with the presence of IgG4 autoantibodies [3, 18, 19]. As IgG4 antibodies form nonprecipitating immune complexes and are not complement-fixing autoantibodies, they do not cause the severe organ damage often seen in hemophilia B patients, in whom allergic reactions to FIX concentrates are associated with the specific IgG1 subclass of alloantibodies against FIX [20].

**Figure 2:**
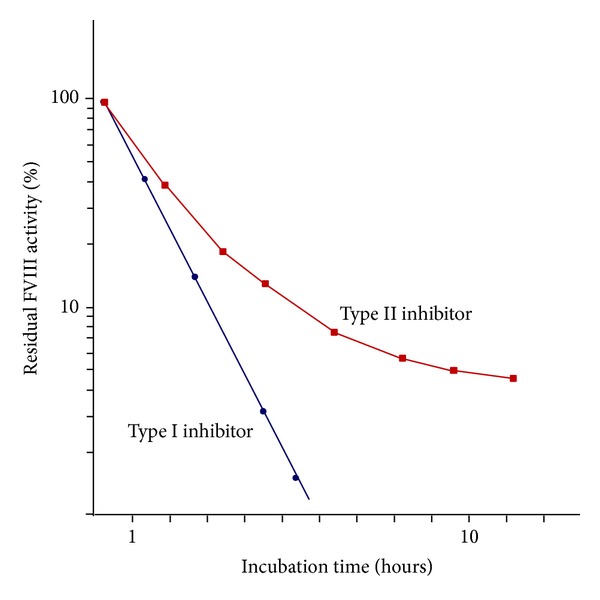
Kinetics of type I and type II inhibitors.

Most alloantibodies developed in congenital hemophilia A patients undergoing FVIII replacement therapy, which are classified as type I inhibitors of first-order kinetics, inactivate FVIII at a rate linearly correlated with their concentration and are able to completely inhibit FVIII activity at high concentrations. In contrast to the kinetics of the interaction between FVIII and the inhibitors in congenital hemophilia A, the kinetics of the interaction in AHA display a nonlinear inhibitory profile. Specifically, these type II inhibitors show a rapid initial inactivation phase followed by a slower equilibrium phase during which some residual FVIII activity (FVIII:C) is detectable even after incubation at maximum concentrations of inhibitors for a sufficient period (Figure 2). However, AHA patients with identifiable FVIII:C manifest far more severe hemorrhage than congenital hemophiliacs with comparable levels of FVIII:C. Moreover, addition of excessive FVIII concentrates fails to neutralize the inhibitory activity of type II inhibitors in vitro, making management of AHA difficult in the clinical setting and high-dose replacement therapy with FVIII concentrates unsuccessful in AHA patients with high-titer inhibitors.

In a study of the physiological activities of AHA inhibitors, Lacroix-Desmazes et al. identified a subset of inhibitors in congenital hemophilia A that hydrolyze FVIII, resulting in FVIII inactivation [21, 22]. On the basis of their findings, Lacroix-Desmazes et al. advocated a unique conception of the inhibitory mechanism in AHA that has been supported by further research of the proteolytic activity of IgG isolated from patients with AHA, demonstrating the presence of autoimmune FVIII-hydrolyzing IgG. On the basis of the observation that the extent of the FVIII hydrolytic activity of acquired inhibitors exhibits a correlation with inhibitory titers of the inhibitors, IgG-mediated FVIII hydrolysis has been hypothesized to participate in FVIII inactivation in AHA [23]. This hypothesis is supported by the results of a comparison study of the properties of the proteolytic inhibitors in congenital hemophilia A and AHA, which, using proline-phenylalanine-arginine-methylcoumarinamide (PFR-MCA), a synthetic substrate for FVIII-hydrolyzing autoantibodies, revealed that the rate of FVIII hydrolysis differs significantly between hemophilia A and AHA patients. While the results of the PFR-MCA and Bethesda assay revealed a correlation between hydrolytic activity and inhibitor titer in acquired inhibitors, alloantibodies in congenital hemophilia A exhibit little correlation. These findings suggest that populations of proteolytic inhibitors in AHA patients differ from those in congenital hemophilia A patients with inhibitors [24]. In addition, some AHA autoantibodies can augment FIX activity by FIX proteolysis in the absence of FVIII-proteolytic activity. On the basis of these findings, it has been hypothesized that the FIX-potentiating action of autoantibodies may partially compensate for the inhibition of FVIII, resulting in restoration of thrombin generation [25].

## 4. Underlying Conditions in AHA

In approximately 50% of AHA patients, especially elderly patients, autoantibody development against factor VIII is idiopathic [2, 26, 27], indicating that the acquired inhibitors develop via an autoimmune mechanism. The underlying conditions shown in Table 1 are observed in the remaining 50% of patients.

### 4.1. Autoimmune Diseases

AHA is often associated with autoimmune diseases, including rheumatoid arthritis, systemic lupus erythematosus, myasthenia gravis, multiple sclerosis, thyroid dysfunction, and autoimmune hemolytic anemia. Observation of an association between AHA and inflammatory bowel disease, pemphigus, and graft versus host disease (GVHD) has been also reported, indicating that AHA has an autoimmune origin. In fact, up to 20% of all AHA patients present with autoimmune disorders [28]. As FVIII inhibitor titers in patients with autoimmune disorders are often high and less frequently resolve spontaneously compared to those associated with pregnancy, the former require aggressive treatment for bleeding management and inhibitor eradication consisting of both hemostatic therapy using bypassing agents and immunosuppressive therapy.

### 4.2. Pregnancy

AHA is associated with pregnancy in approximately 10% of cases [8]. Although hemorrhagic symptoms commonly present between 1 and 4 months after parturition, they may occur over a year after delivery [29, 30]. While the hemorrhagic potential is often low and the inhibitors often spontaneously disappear in almost all patients with low titers of inhibitors [29], it may be difficult to achieve inhibitor eradication in patients with high titers (≥5 BU/mL), even with aggressive immunosuppressive therapy. An important consideration is that carrying a fetus might pose the risk of fatal bleeding, as it poses the risk of diaplacental transition of inhibitor IgG from pregnant AHA patients [30]. When inhibitor eradication in patients with postpartum inhibitors is unsuccessful, other commonly associated conditions, especially autoimmune disorders, should be suspected.

### 4.3. Malignancy

Underlying malignancy in either solid or nonsolid form presents in approximately 10% of AHA patients and commonly develops in elderly patients. An important consideration is that, as the incidence of both solid tumor as well as AHA increases with aging, inhibitors might be detected coincidentally in patients with solid tumor. Patients with lymphoproliferative diseases complicating AHA, which include chronic lymphocytic leukemia, non-Hodgkin lymphoma, and multiple myeloma [8], with altered immune status often have coexisting autoimmune diseases, one of which may be AHA. As anticarcinogenic agents induce cell damage and/or modulate immunological reactions through danger signals [31, 32], patients with malignancies might be predisposed to autoimmune phenomena and increased risk of developing inhibitors. Although it remains unclear whether autoantibody development derives from the tumor itself, the observation that cancer antigens share immunological cross-reactivity with FVIII has not been reported to date.

### 4.4. Medical Agents

Reactions associated with drug hypersensitivity have been implicated in the onset of AHA. Suspected medications include antibiotics (penicillin, sulfonamides, and chloramphenicol), anticonvulsants (phenytoin), antihypertensive agents (methyldopa), and bacillus Calmette-Guérin vaccination [2]. As high titers of inhibitors resulting from drug reactions and allergies disappear after termination of the responsible drug [29], specific therapy to eradicate FVIII autoantibodies may not be provided to patients who experience drug hypersensitivity. It is notable that administration of interferon for hepatitis C virus infection, which, by directly acting on the immune system, results in malfunctioning of the immune response, is associated with AHA [33].

## 5. Molecular Biological Mechanisms

Anti-FVIII autoantibodies are developed in the context of dysfunction of immune system, as discussed above. Knowledge of the detailed molecular biological mechanism of inhibitor generation has accumulated gradually over the past decades.

### 5.1. CTLA-4

Variants of the polymorphic cytotoxic T lymphocyte antigen-4 (CTLA-4) gene, which is found on the surface of activated and regulatory T-cells, have been associated with autoimmune diseases [34]. The extracellular domain of CTLA-4 is similar to the domain of the CD28 that is a component of the costimulatory CD28/B7 receptor/ligand system and competes against CD28 ligands, such as CD80 and CD86, on the surface of dendritic cells. Stimulation of the CD28 receptor on T-cells emits a costimulatory signal for T-cell proliferation and activation. In contrast, CTLA-4 may inhibit T-cell activation by restricting the ability of B7 to interact with CD28 [35]. In regulatory T-cells, CTLA-4 is constitutively expressed at a steady state through transcriptional enhancement by Foxp3. Tight binding of CTLA-4 molecules on regulatory T-cells to costimulatory ligand B7 on antigen-presenting cells strips and destroys B7 molecules. As antigen-presenting cells without B7 ligands cannot deliver additional Signal 2 (i.e., engage in costimulation), the binding of CTLA-4 and costimulatory ligand B7 terminates activation or differentiation of naïve T-cells to effector T-cells. Thus, CTLA-4 acts as a receptor that downregulates the immune system. The results of several studies, including those of a recent study that observed a single nucleotide polymorphism of the CTLA-4 gene (+49 A/G allele) at a significantly higher frequency in AHA patients compared with controls [36], indicate that CTLA-4 variants (CTLA-4 single nucleotide polymorphisms) might also be involved in the pathogenesis of AHA as well as that other genetic/environmental factors might contribute to the onset of AHA.

### 5.2. BAFF

Recently, B-cell activating factor belonging to the tumor necrosis factor family (BAFF), also referred to as BlyS, has been found to regulate the immune system. Known to be involved in the survival and maturation of B-cells [37], BAFF binds to tumor necrosis factor-related receptors, such as B-cell-maturation antigen (BCMA), transmembrane-activator and calcium-modulator and cyclophilin-ligand interactor (TACI), and B-cell activating factor receptor (BAFF-R) [38]. This BAFF-mediated ligand-receptor interaction forms a complex network that plays a critical role in the induction and regulation of humoral immunity. Previous mouse studies demonstrated that constitutive BAFF overexpression leads to survival of autoreactive B-cells [39], which in turn induces breakdown of peripheral tolerance. In this setting, autoimmune disorders develop through anomalous B-cell activation with spontaneous production of multiple autoantibodies and polyclonal hypergammaglobulinemia. In humans, elevated BAFF levels are associated with several B-cell-mediated autoimmune diseases with hypergammaglobulinemia [40–42].

In a previous study, we found BAFF levels to be significantly higher in congenital hemophilia A patients with inhibitors compared to healthy controls or hemophilia A patients without inhibitors [43]. These results suggest that elevated BAFF levels allow anti-FVIII antibody-secreting plasma cells to survive and produce inhibitors in congenital hemophilia A patients with inhibitors. Despite such research, the typical presentation of BAFF levels in patients with AHA remains to be elucidated. Our preliminary measurement of BAFF in two patients with AHA revealed an elevated level of BAFF in one patient but a normal level in the other, suggesting that BAFF might be involved in the pathogenesis of AHA in at least some AHA patients. Although further study is warranted before its application in the clinical setting, the targeting of BAFF as a therapeutic strategy appears promising in the treatment of a subset of AHA patients, as well as of hemophilia A patients with refractory inhibitors presenting with elevated BAFF levels.

## 6. Diagnosis

### 6.1. Cross-Mixing Testing

The first step in diagnosis of AHA is tracking signs of bleeding tendency, particularly in the elderly, in the clinical setting and testing for prolongation of activated partial thromboplastin time (APTT) in the laboratory. The next step is review of patient medical history by consideration of the impact of any underlying conditions associated with AHA. APTT prolongation reflects decreased levels of coagulation intrinsic factors VIII and IX, as well as decreased levels of factors XI and XII, prekallikrein, and high molecular weight kininogen, which are involved in the contact system of coagulation. However, since reduction of proteins involved in the contact system is not associated with bleeding tendencies [44, 45], these conditions are ruled out in the differential diagnosis.

To diagnose AHA, measurement of FVIII:C is essential, and consecutive determination of inhibitor titer is a requisite in cases of decreased level of FVIII:C. While APTT is prolonged in patients with low levels of FVIII:C by anti-FVIII neutralizing autoantibodies, PT, fibrinogen and VWF levels, and platelet count are within normal limits and platelet function is normal. Since thrombocytopenia, PT and APTT prolongation, and decreased levels of fibrinogen are often observed in DIC patients who are erroneously diagnosed with AHA, consideration of these findings is helpful in differentiation of DIC from AHA.

**Figure 3:**
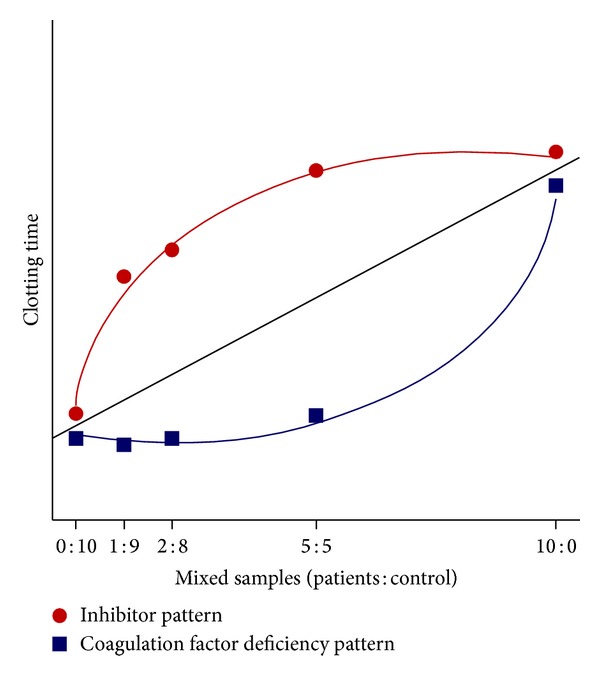
Cross-mixing test for detection of lupus anticoagulants or inhibitor of coagulation factor. A convex upward curve indicates the presence of inhibitors, including lupus anticoagulants and coagulation factor-neutralizing antibodies, while a convex downward curve indicates the presence of a factor deficiency.

Several cross-mixing studies have been performed to examine whether APTT prolongation results from a deficiency of intrinsic factor(s) or inhibitor. In one such study, addition of an equal volume of normal control plasma to patient's plasma was found to correct the APTT value to the normal range in coagulation-factor-deficient patients but not AHA patients [46]. As FVIII inhibition by autoantibodies is time- and temperature-dependent, the mixture in all such studies should be incubated at 37°C for 1 to 2 hours and, if correction of APTT value is unsuccessful, the presence of an inhibitor should be suspected. Recently, a cross-mixing test originally developed to differentiate lupus-anticoagulant presence from coagulation-factor deficiency has been established as a more useful laboratory test to determine the cause of APTT prolongation and thus useful in AHA diagnosis [47]. The plotting of the results of a cross-mixing test with altering the proportion of normal control plasma to the patient's plasma yields a convex APTT value curve that faces upward in the presence of inhibitors (including coagulation factor-neutralizing antibodies and lupus anticoagulants) and downward in the presence of a factor deficiency (Figure 3).

### 6.2. Lupus Anticoagulant

APTT is prolonged in the presence of lupus anticoagulants that interfere with the assembly and activity of the FXa-FVa-Ca^2+^ phospholipid complex. Lupus anticoagulants are polyclonal immunoglobulins that bind to phospholipids and proteins associated with the cell membrane and show nonspecific inhibitory effects that result in prolongation of both APTT and PT. From the perspective of laboratory testing, since intrinsic coagulation factor activity, including that of FVIII, appears to decrease in the presence of lupus anticoagulants, it is often difficult to distinguish AHA from lupus anticoagulants even by a mixing test. If the results of a mixing test indicate the presence of an inhibitor, the lupus anticoagulant is therefore evaluated by phospholipid-sensitive functional-coagulation assay, such as the dilute Russell's viper-venom time assay [48]. The coagulant in the venom directly activates FX, indicating that the dilute Russell's viper-venom time assay is dependent on a common pathway, including a pathway with phospholipids, and not influenced by deficiency or inhibition of intrinsic factors. Thus, the addition of exogenous phospholipids will correct the prolongation value as measured by clotting assay, confirming the presence of a lupus anticoagulant. One type of clotting test, the platelet neutralization procedure (PNP), takes advantage of the fact that lupus anticoagulants are absorbed onto the phospholipids on the surface of platelets while FVIII inhibitors are not absorbed [49]. The addition of washed platelets to the patient's plasma with lupus anticoagulant will thus decrease APTT prolongation.

### 6.3. Inhibitor Measurement

When the presence of an inhibitor is suspected, the targeted factor should be identified and the extent of inhibitory activity quantified. For the quantification of FVIII inhibitors, the Bethesda assay is the most commonly used laboratory test worldwide [50]. The classic Bethesda method measures the quantity of residual FVIII:C of a mixture containing equal amounts of normal control plasma and serially diluted patient plasma after incubation at 37°C for 2 h. The level of residual FVIII:C in the patient's plasma with inhibitors increases in tandem with the increasing dilution rate. The inhibitor titer value (1.0 BU/mL) used in the Bethesda assay is the reciprocal of the value of the dilution of the patient's plasma that leads to 50% inhibition. In the Nijmegen modification, which permits more accurate measurement of low titers of FVIII inhibitor, buffer is added to the Bethesda assay to maintain the sample plasma pH within the physiological range for the 2-hour incubation period and thereby stabilize FVIII in normal control plasma [51].

**Figure 4:**
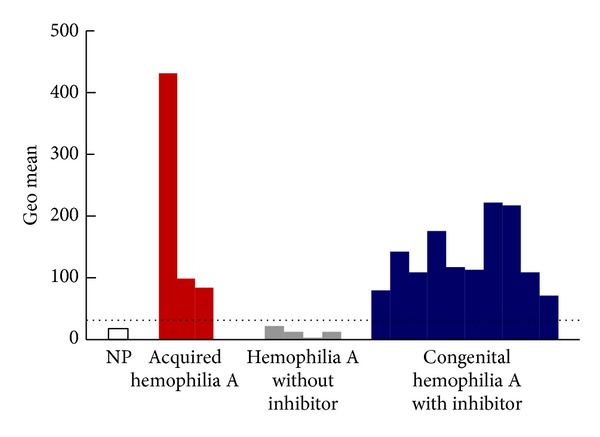
Flow cytometric analysis of factor VIII- (FVIII-) binding antibodies. Plasma samples from 20 normal healthy volunteers (normal pooled plasma), 3 acquired hemophilia A patients, 4 congenital hemophilia A patients without inhibitors, and 10 congenital hemophilia A patients with inhibitors were assessed using the following procedure. Human recombinant FVIII (rFVIII) was bound to red fluorescent carboxylated polystyrene microbeads (Cyto-Plex polystyrene microbeads) and a certain number of human rFVIII-bound microbeads were added to serially diluted suspected plasma. After incubation and washing, PE-labeled anti-human IgG antibody was added to the microbeads. After additional incubation and washing, fluorescent intensity was measured using a FACScan flow cytometer. The fluorescence intensity of the anti-human IgG antibody bound to human rFVIII on the microbead surface was expressed as the geometric mean (shown in arbitrary units). The dotted line shows a tentative cutoff value for the inhibitor with the highest geometric mean value in plasma without inhibitor. NP: normal plasma.

Although these assays are useful for determination of titers of alloantibodies against FVIII in congenital hemophilia A patients with type I kinetics, exact determination of autoantibody titer in AHA is difficult in patients with type II kinetics, in whom the acquired inhibitor-FVIII complex may show some residual FVIII:C, even in the presence of high concentrations of inhibitors. Therefore, measurement of levels of FVIII-binding antibodies is necessary for performing meaningful clinical assessment of the inhibitors present in AHA [13]. Prior to development of enzyme-linked immunosorbent assay (ELISA), measurement of FVIII-binding antibodies was traditionally performed using the agarose gel method [52, 53]. Previous studies have demonstrated that noninhibitory antibodies can be detected by ELISA in hemophilia patients in whom no inhibitors were detected using the Bethesda method [54–57]. In accordance with previous research into the use of the immunoprecipitation method for measurement of noninhibitory antibodies [58, 59], our investigation of the efficacy of immune-tolerance induction therapy in hemophilia A with refractory inhibitors using the immunoprecipitation method revealed that the method yields results of sufficient sensitivity [60]. However, use of all of these methods has certain drawbacks, such as the need to use radioactive materials and perform complex, time-consuming procedures. Fortunately, the ability of fluorescent microbeads method to overcome these drawbacks has been demonstrated in several studies [61], including one study in which we demonstrated its usefulness for assessing AHA patients as well as hemophilia A patients with inhibitors (Figure 4) [62]. As measurement using the fluorescent microbeads method is almost completely unaffected by the presence or absence of residual FVIII:C, use of the method allows for detection of antibodies without the undue influence of the presence of lupus anticoagulants or heparin.

## 7. Clinical Management

Favorable outcome in AHA depends on selection of an appropriate therapeutic approach based on early, correct diagnosis. The therapeutic strategy should aim for the achievement of 2 targets: control of bleeding and eradication of inhibitors.

### 7.1. Treatment of Acute Bleeding

Bleeding episodes in AHA are often severe and life threatening and presents with severe anemia. As massive subcutaneous or intramuscular hemorrhage may continuously worsen if left untreated, provision of immediate hemostatic therapy and monitoring of its efficacy by observation of improvement in anemia and clinical manifestations is required. The first-line treatment for severe bleeding episodes, especially in patients with high titers of inhibitors, is administration of bypassing agents [63, 64]. Activated prothrombin complex concentrates (APCC) containing factors II (prothrombin), VII, IX, and X or recombinant activated factor VII are commonly administered and have shown to be beneficial in treating patients with AHA as well as congenital hemophilia A patients with inhibitors [63–66]. Use of the immunoadsorption technique for removal of high-titer inhibitors has also proven beneficial in AHA patients with acute, life-threatening bleeding [67].

Another hemostatic treatment, the provision of inhibitor-neutralizing therapy with administration of FVIII concentrates at a level sufficient for neutralizing inhibitors, may also be beneficial for these patients. However, it is difficult to determine the quantity of FVIII required and calculate the half-life of the infused FVIII owing to the presence of type II inhibitors in AHA. In contrast, the requisite quantity of FVIII for neutralizing type I inhibitor in congenital hemophilia A can be determined theoretically. Therefore, frequent monitoring of hemostatic functioning accompanied by measurement of FVIII:C and/or APTT should be performed while providing neutralizing therapy to AHA patients. Administration of desmopressin, which stimulates the release of FVIII and VWF from endothelial cells and can provide a transient rise in FVIII:C levels to therapeutic levels [68], may also be effective in AHA patients with low titers of inhibitors or an FVIII:C level >5% [64]. While desmopressin has the advantages of being of low cost and safety, it does not entirely increase FVIII:C level to a therapeutic level and becomes less efficacious with repetitive administration. As neither therapy is adequate for AHA patients with high titers of inhibitors or severe bleeding symptoms, a bypassing strategy should be used with these patients.

### 7.2. Suppression of Inhibitor Formation

As with congenital hemophilia A with inhibitors, suppression and eradication of inhibitors are essential for normalization of hemostatic function and elimination of the risk of hemorrhage in AHA. For this, provision of immunosuppressive therapy is critical. In some cases of postpartum and drug-induced acquired hemophilia that resolves spontaneously, immunosuppressive therapy may be unnecessary [69]. However, even if bleeding symptoms are mild, the risk of severe and fatal hemorrhage persists unless inhibitors are eradicated. Therefore, immediate initiation of immunosuppressive therapy after confirmation of AHA diagnosis is recommended [70–73]. Several studies have established the effectiveness of immune-tolerance-induction therapy based on repetitive high-dose FVIII infusion for the eradication of inhibitors developed in congenital hemophilia A [74]. Immune-tolerance-induction methods that have been reported to be effective for treating AHA include not only administration of high-dose FVIII but also immunoadsorption and immune suppression therapy [75], the latter of which is likely essential for therapeutic success.

Agents used in immunosuppressive therapy for suppression of inhibitors include immunosuppressive agents such as prednisone, azathioprine, and cyclosporine and antineoplastic agents such as cyclophosphamide (CPA), mercaptopurine, and vincristine. Among these, administration of prednisone alone or in combination with CPA has been a common strategy. Combined prednisone-CPA administration has been reported to yield favorable outcomes [29, 64, 76], indicating that combined use of immunosuppressive or antineoplastic agents and prednisolone may yield beneficial effects. High-dose intravenous immunoglobulin therapy can be provided as an adjunctive therapy but should not be used as an initial treatment [12]. Physical removal of inhibitors by plasma exchange therapy or protein A adsorption column is effective for transient removal of inhibitors in patients with acute, severe bleeding [69]. Recently, several case studies of successful treatment with chimeric monoclonal antibodies targeted against the pan-B-cell marker CD20 (rituximab) in patients refractory to initial immunosuppressive therapy have been reported [77]. In cases where increased BAFF levels activate B-cells, use of a strategy to suppress B-cell activation appears rational.

There is no evidence that one immunosuppressive therapy is clinically superior to all others in treating AHA or that a certain therapy should be chosen depending on inhibitor titer or the hemorrhagic status. Therefore, first-line treatment is determined by evaluation of disease condition and consideration of possible adverse effects [64]. Although acute hemorrhage in AHA is potentially lethal, infectious diseases, such as pneumonia and sepsis, are responsible for approximately 50% of mortality associated with AHA [78]. Therefore, sufficient attention to prevention and early detection of infectious disease is warranted when aggressive and prolonged immune suppression therapy is provided.

## 8. Conclusions

AHA is characterized by the presence of an autoimmune mechanism that alone or accompanied by autoimmune disease, aging, pregnancy, or drug exposure causes breakdown of immune tolerance to FVIII associated with CD4 T-cells and results in development of autoantibodies against FVIII. In addition to treatment for acute bleeding, which is often required for AHA patients, immune suppression is essential for eradication of the inhibitors that play a central role in AHA pathogenesis. While provision of immunosuppression therapies, such as combined prednisone-CPA therapy, is currently the first-line treatment, administration of anti-CD20 monoclonal antibody (rituximab) appears to be a promising alternative treatment for AHA. Consideration of the findings regarding the association between the autoimmune mechanism responsible for AHA development and the innate immune system presented here and further elucidation of this association in future research will provide for better understanding of AHA pathophysiology and the development of novel therapies for eradication of inhibitors.

## Figures and Tables

**Table 1:** Conditions associated with acquired hemophilia A.

Idiopathic	Malignancy
Autoimmune diseases	Squamous cell cancer
Rheumatoid arthritis	Lymphoproliferative diseases
Systemic lupus erythematosus	Chronic lymphocytic leukemia
Myasthenia gravis	non-Hodgkin lymphoma
Multiple sclerosis	Multiple myeloma
Thyroid dysfunction	Medical agents
Autoimmune hemolytic anemia	Antibiotics
Inflammatory bowel diseases	Penicillins
Pemphigus	Sulfonamides
Graft versus host disease	Chloramphenicol
Pregnancy	Anticonvulsants (phenytoin)
	Antihypertensive (methyldopa)
	Bacillus Calmette-Guérin vaccination
